# Supplementary material for: Patterns of Intron Gain and Loss in Fungi
Source: PLoS Biol. 2004 Nov 30;2(12):e422. doi: 10.1371/journal.pbio.0020422 (PMC532390; doi:10.1371/journal.pbio.0020422)
Supplement: Table S1 — Also available at http://genes.mit.edu/NielsenEtAl/. (4.3 MB ZIP). [file pbio.0020422.st001.zip › NielsenEtAl/html/720.html]

AN4456.1.NCU01367.1.MG07030.1.FG06802.1


```
 CLUSTAL W (1.82) Multiple Sequence Alignments - Introns Inserted


Sequence 1: NCU01367.1	629 aa
Sequence 2: MG07030.1	609 aa
Sequence 3: FG06802.1	587 aa
Sequence 4: AN4456.1	625 aa
Alignment Length: 692 aa
Number Identitical Residues: 194 aa
Alignment Score (without introns) 11197


MG07030.1 	MSSFFTVP-GAQKRKRTGAPENPKKRLATSGSRPSKAQPSKSRDKASSAAPAKKRVERD-
NCU01367.1	MSSFFTAPAGEKKRKRASANEAPQKRVAT-GKSTAKSSSVRGPAAKSRPAAPKKAVERD-
FG06802.1 	MSSFFTVPGAQKKRKRPEASEQPKKRVAA--------SKSAGKAPSKNAGPTKKRVERDD
AN4456.1  	MSSFFTLPASQRKRKREDRAGAPASKKRG--------VDVDGDARNKN---ARKSRERD-
          	****** *.. :****      * .:               .    .    .:*  *** 

MG07030.1 	ESISGSDSDSDNDQDQDADDFEAGSGSDDDSEREGETAAERRLRLAERYLDNVRQEVAAE
NCU01367.1	ESISGSDIDSD---DGDHIEREDGSEGEEESDHEGETAAEKRLRLAQRYLDKARQEVELE
FG06802.1 	ESISGSDSDDDGSLDSASVASDVEMEDNESEDEEGETARDKRMRLAERYLENIQQTV---
AN4456.1  	ESISGSDLDED----AESVASVVSEEESGSESDDGETAADRRLKLAERYLENVREEV---
          	******* *.*                . ... :**** ::*::**:***:: :: *   

MG07030.1 	DEYAFDAEEIDRDIIAERLKEDV~AESKGKVYRHVARELDYPKTYQTGFRWNPENVTGVA
NCU01367.1	DEYAFDAEQIDRDLLAERLQEDV~AEAKGKVYRQLASKLDFDKAAHTQFRWNSGTVTSVS
FG06802.1 	DEAGFDAADVDRDLMAERLQEDV~AEGKGRVYRQLASEFAFDNVSRTFFRSNTNAVTAIA
AN4456.1  	DDYGFDAAEIDRDLIAERLKEDV0DEFKGRVYRQIASDLAFSTASHAFFRADTQTTTSIA
          	*: .*** ::***::****:***  * **:***::* .: : .. :: ** :.  .*.::

MG07030.1 	TCPPYAYLVTKDMFLSKWKLQDLPQYQWPQTT--RKKSKKPNAPPKRRPEKVASLRGDSR
NCU01367.1	VCAPYAYTTTKDGYLTKWKLQDLPKNQWPQTT--KKKPKKPPAPPKKKPERVCFNRANPI
FG06802.1 	VCDPYIYTTTKDLYLHKWKAQNLPQYQYPQTT--RRKPKKPPAPPKKQPSLEAWVKGDTR
AN4456.1  	IHAPYIYTVSKDRTLIKWELATPGAAGTATTNGAENTSKRPPKPQRKKPKQVRFTRGLQK
          	   ** * .:**  * **:         . *..:....*:*  * :::*.     :.   

MG07030.1 	KASDKSYHGHT-GAILAVAASQDGKWVVTAGADKRIVVHDAQTLKPVRALTHHRDAVTSL
NCU01367.1	KAKDKTYQGHTNAAILTVRASADGKFVVTGGADKRMVVHDAETLKPLRAFTQHRDAVTSL
FG06802.1 	KSKDKNYQRHT-DRILAVAASPDGKYVATGGADNKLIIYDAHTLKPIKIWTHHRDSVTGL
AN4456.1  	VAESADEQGHT-KNILSVAVSPSGRFVATGGEDNKLIIWDAATLTPLQTFSQHRDSVSGL
          	 :..   : **   **:* .* .*::*.*.* *::::: ** **.*::  ::***:*:.*

MG07030.1 	AFQR------GTNQLYSGSRDRTVKVWNIDNL--AYIETLFGHQDHVVDIDALAQERCVS
NCU01367.1	AFRR------GTNQIFSASKDRTVKVWSLDEL--AYVETLFGHQDEIPDIDALAGERCVS
FG06802.1 	VFRR------GTNQLYSCSKDRTVKVWSLDEM--AYVESLFGHQDEILDVDALAQERCVS
AN4456.1  	AFARHISAMSSGEQLFSGSFDRTIKTWSISTAGHAYVETLFGHQDNVASVAAMTIDQCIS
          	.* *  :: :. :*::* * ***:*.*.:.  . **:*:******.: .: *:: ::*:*

MG07030.1 	AGARDRTARLWKVVEESQLVFRGGGGTTLNKKNNCPEGVDPKSLAHEGNVDRIAMLDDEL
NCU01367.1	VGARDRTARYWKVPEESQLVFRGGVSEKKSHKN------RDQAVNHDGTMDQVAMIDDEL
FG06802.1 	VGARDRTARLWKVAEETQLVFRGGAVDKKSRPS----GVSARSMLHEGSMDRVAMIDDDL
AN4456.1  	VGARDRTARLWKVVEESQLIFRGGSSKNTYKEN---------------NIDCIAPLPPTH
          	.******** *** **:**:****   .  : .               .:* :* :    

MG07030.1 	FVTGSDNGSISLWSLQKKRPVFVLPLAHGLEPPLPLEAASADKSPDPKVIPP----PQPR
NCU01367.1	FVTGDSAGTLSLWGINRKKALFTQPCAHGIDPPLKPTEVSADANPDESVVPT----PQPR
FG06802.1 	FVTGSDNGALALWSVQKKKPVYIEPIAHGIDPALDPKEASAEQNPDPAIVPA----PTPR
AN4456.1  	FVTGSDNGSISLWSIHKKKPLYTIPHAHGFDPIPPVELISPEVDQQTAESNARFLRPTPR
          	****.. *:::**.:::*:.::  * ***::*       *.: . :     .    * **

MG07030.1 	WITALRTIPYSDLILSGSWDGCVRIWRLSDDKKKLEAAGVLG-----EPLEQSQD-----
NCU01367.1	AITALRTIPYSDVILSGSWDGFVRVWRLSEDKKKILPVGVLGSSTALQQLDQAEAQKQKE
FG06802.1 	WITAIRTIPYSDVILTGSWDGHIRVWQLSEDKRKIEFRGVLS------------------
AN4456.1  	WITALTTVPGTDIVLSGSWDGWIRAWKISDDKRTIQPLGPVG-----AGTYRSTN-----
          	 ***: *:* :*::*:***** :* *::*:**:.:   * :.          :       

MG07030.1 	-------------TSASDKDKSNGKAITNGNSS---------HSKLIKGIVNDLSVFER-
NCU01367.1	EAKQKAKAEKEPATASSGEEQADDEASVDGSQDKK----KKQESGLIRGIINDIAIFER-
FG06802.1 	--------------KREDQEQESTEANAQGPIN---------------GVVNDIDLFER-
AN4456.1  	----------APDTPSKQLEQSLASANPSHRQSLTNINFEPESEPLVKGVVNGIAVFERR
          	           . :  .  ::   .*  .   . .. . . . .    *::*.: :*** 

MG07030.1 	GDRGKDG-------------------ICVVAAVGKEHRLGRWKR---------IKGGQNG
NCU01367.1	GNRGRDG-------------------ICIVVATGKEMRLGRWKY---------MKEGRNG
FG06802.1 	GDRGKDG-------------------LCVVVAVGKEHRLGRWNPP--------AKIGRNG
AN4456.1  	AETAKPGQVPSESKSKPSKTSPEARGLCIAVAIGKEHRFGRWKCYANNYHKGSASDGRNG
          	.: .: *. .:.:.:..:.::..: .:*:..* *** *:***:  :..  ..: . *:**

MG07030.1 	AFVFEVPKIVKEKLALNGT---SHTSDDGDE--
NCU01367.1	ATIFEVPKVARRAGAKGEE---SDEEEEEEE--
FG06802.1 	GVVFEIPRIQRQTVVNGTNENGTNESSDGEQDE
AN4456.1  	AMVFEVPFIAGSVGQEDEP---SN---------
          	. :**:* :       .     :.
```
